# From mitochondria to sarcopenia: role of 17β-estradiol and testosterone

**DOI:** 10.3389/fendo.2023.1156583

**Published:** 2023-04-20

**Authors:** Xu Tian, Shujie Lou, Rengfei Shi

**Affiliations:** School of Kinesiology, Shanghai University of Sport, Shanghai, China

**Keywords:** skeletal muscle, mitochondria, sarcopenia, 17β-estradiol, testosterone, aging

## Abstract

Sarcopenia, characterized by a loss of muscle mass and strength with aging, is prevalent in older adults. Although the exact mechanisms underlying sarcopenia are not fully understood, evidence suggests that the loss of mitochondrial integrity in skeletal myocytes has emerged as a pivotal contributor to the complex etiology of sarcopenia. Mitochondria are the primary source of ATP production and are also involved in generating reactive oxygen species (ROS), regulating ion signals, and initiating apoptosis signals in muscle cells. The accumulation of damaged mitochondria due to age-related impairments in any of the mitochondrial quality control (MQC) processes, such as proteostasis, biogenesis, dynamics, and mitophagy, can contribute to the decline in muscle mass and strength associated with aging. Interestingly, a decrease in sex hormones (e.g., 17β-estradiol and testosterone), which occurs with aging, has also been linked to sarcopenia. Indeed, 17β-estradiol and testosterone targeted mitochondria and exhibited activities in regulating mitochondrial functions. Here, we overview the current literature on the key mechanisms by which mitochondrial dysfunction contribute to the development and progression of sarcopenia and the potential modulatory effects of 17β-estradiol and testosterone on mitochondrial function in this context. The advance in its understanding will facilitate the development of potential therapeutic agents to mitigate and manage sarcopenia.

## 1 Introduction

Sarcopenia is a skeletal muscle disorder characterized by progressive and generalized loss of muscle mass and strength/function upon aging, with an increased risk of adverse outcomes, including falls, disability, loss of independence, morbidity, and mortality (1). Muscle mass and strength peak in young adulthood and, after a plateau, start decreasing gradually with advancing age (2, 3). Such a decline can be accelerated in persons with inactive lifestyles (4), as well as in the setting of chronic diseases (5) or inadequate nutrition (6). The sarcopenia process is accompanied by progressive muscle wasting attributed to increased levels of apoptosis (7) and reduced capabilities for muscle regeneration (8). This cellular and functional deterioration is a multifactorial etiology, in which hormonal alterations (9), neuromuscular junction degeneration (10), muscle fat infiltration (11), oxidative stress (12), chronic inflammation (13), and mitochondrial dysfunction (14) are the crucial factors accountable for this pathology. However, the specific role of these factors and the exact molecular mechanisms triggered by those conditions are not fully understood.

In humans, skeletal muscle is one of the most dynamic and plastic tissues in the body, comprises approximately 40% of total body weight, and contains 50–75% of all body proteins (15). Skeletal muscle is made up of bundles of muscle fibers (muscle cells) named fascicles. The cell membrane surrounding the muscle fiber is the sarcolemma, and beneath the sarcolemma lies the sarcoplasm, which contains the cellular proteins, organelles, and myofibrils: the thin filaments (actin) and the thick filaments (myosin). Actin and myosin are contractile proteins that are responsible for muscle contraction. Mitochondria form a three-dimensional network in the sarcoplasm that produces the energy needed for muscle contraction when oxygen is available to the muscle fibers (16). Besides, efficient communication between the nervous and muscular systems is a critical factor in the ability of muscle contraction. Muscle fibers connect with motor neurons to form the neuromuscular junction (NMJ), a highly specialized synapse. There, neuronal signals from the brain or spinal cord are delivered to the sarcolemma *via* the neurotransmitter acetylcholine, triggering the depolarization of muscle fibers and initiating muscle contraction. Hence, the maintenance of functioning muscle mass is a complex process, and malfunction of any of the above elements can lead to muscle deterioration.

Emerging evidence suggested that apart from energy production, mitochondria perform other critical functions to maintain homeostasis and function in the skeletal myocytes, including regulation of intracellular Ca^2+^ homeostasis, modulation of cell proliferation, and integration of apoptotic signaling (17–19). Aberrant mitochondrial quality control (MQC) leads to loss of mitochondrial integrity, which is thought to be a major factor in muscle degeneration (20). Along with mitochondrial alteration, age-related decreases in sex steroid hormones in both men and women have been implicated as critical factors in the development of sarcopenia (21, 22). Notably, mitochondria have sex-specific features, and alterations in mitochondrial quality control are associated with changes in sex steroid hormone levels (23, 24). Here, we will outline the current understanding of the central mechanism by which mitochondria contribute to skeletal muscle health during aging and the potential correlation between the mitochondria and sex hormones in the pathogenesis of sarcopenia, focusing on recent findings.

## 2 Mitochondria structure and function

### 2.1 Mitochondria and mitochondrial life cycle

The mitochondrion is a micron-sized, densely arranged, dynamic organelle that exists in cells as granular or filamentous structures and varies in number across tissues or cell types. According to the endosymbiotic hypothesis, mitochondria were initially derived from aerobic α-proteobacteria, which, over billions of years of evolution, were engulfed by anaerobic archeobacteria to form primitive eukaryotic cells (25). Due to their ancient bacterial origin, the bilayer membrane of mitochondria also seems to be explained accordingly. The inner membrane is rich in cardiolipin, which is thought to be derived from the cell membrane of the bacteria itself, while the outer membrane is originated from the cell membrane of the host cell. These two membranes divide the mitochondria into two compartments: the intermembrane space and the matrix space. The inner mitochondrial membrane folds inward to form the mitochondrial cristae, responsible for more biochemical reactions that convert dietary fuel metabolites into ATP, CO_2_, and H_2_O to sustain life.

Unlike other organelles in cells of the human body, mitochondria harbor a small amount of their own DNA (mtDNA), which encodes a series of critical proteins involved in mitochondrial respiration, including 13 genes encoding subunits of the respiratory chain, two genes encoding ribosomal RNAs, and 22 genes encoding transfer RNAs (26). Similar to bacterial chromosome, mtDNA is packaged by a series of proteins, including prohibitins, ATPase family AAA domain-containing protein 3 (ATAD3), the mitochondrial transcription factor A (TFAM), and mitochondrial polymerase gamma catalytic subunit (POLG) to form mtDNA-protein complexes called nucleoids (27, 28). TFAM is known to be the main nucleoid protein, acts as a transcription factor for mtDNA, and plays an important role in nucleoid compaction and mtDNA maintenance (27). Genetic defects of mtDNA are associated with many human diseases (29). Different from nuclear DNA (nDNA), mtDNA is inherited only through the maternal line, is intronless, and lacks histones (30). All other mitochondrial proteins, including those involved in mtDNA replication, repair, transcription, and protein translation, are nuclear-encoded and translocated to the mitochondria using specialized import systems — the translocase of the outer membrane (TOM) and translocase of the inner membrane (TIM) complexes (31). The TOM complex recognizes and binds to the mitochondrial targeting sequence of the incoming protein, while the TIM complex facilitates the translocation of the protein across the inner mitochondrial membrane (32).

Sufficient energy supply and cellular survival rely upon the complex mitochondrial life cycle, which involves mitochondrial biogenesis, fission-fusion, and mitophagy (33). Mitochondrial biogenesis and degradation(mitophagy) are a set of opposing processes to maintain a dynamic equilibrium of cellular mitochondrial content. Mitochondrial proliferation is achieved by increasing nuclear and mitochondrial-encoded proteins, mtRNA, and other components (i.e., mitochondrial biogenesis), followed by mitochondrial fission. Regulation of mitochondrial biogenesis varies among tissues and stimulation factors but often involves some important co-activators and specific transcription factors, including the peroxisome proliferator-activated receptor γ co-activator 1-α (PGC-1α), nuclear respiratory factors (NRF1 and NRF2), estrogen-related receptors (ERRα, ERRβ, and ERRγ), and TFAM (34). Dysfunctional and damaged mitochondria can be effectively degraded, eliminated, and recycled *via* mitophagy. This process is regulated by some proteins, among which the PTEN-induced putative kinase 1 (PINK1) and E3-ubiquitin ligase Parkinson juvenile disease protein 2 (PARK2) are served to recognize damaged mitochondria and localize them to the autophagosome, which then fuses with lysosomes to form autophagolysosomes (35, 36).

Just as mitochondrial biogenesis and mitochondrial autophagy dynamically regulate mitochondrial quantity and quality, mitochondrial fusion and fission serve as another set of opposing processes to regulate mitochondrial production and morphology. Mitochondrial fission separates the damaged part from the healthy mitochondrial network or occurs during cell mitosis, producing two new mitochondria to meet cell division needs (33, 37). A few mitochondrial outer membrane proteins, including mitochondrial parting protein 1 (FIS1), mitochondrial splitting component (MFF) (38), and mitochondrial dynamics proteins of 49 kDa and 51 kDa (MiD49, MiD51) (39), are identified to mediate mitochondrial fission by targeting the GTPase dynamin-related protein 1 (DRP1) to the mitochondrial surface. On the other hand, fusion allows the transfer of metabolites, enzymes, and gene products between mitochondria for optimal functioning. Mitochondrial fusion is mediated by three large GTPases of the dynamin superfamily: Mitofusin 1 (MFN1), Mitofusin 2 (MFN2), and optic atrophy 1 (OPA1) (40).

### 2.2 Mitochondrial functions

As the cell’s powerhouse, mitochondria primarily use fatty acid and carbohydrate-deriving substrates to generate reducing equivalents, eventually converted to chemical energy in the form of ATP. The common degradation product of fatty acids and carbohydrates in mitochondria is acetyl-CoA, which undergoes a series of enzyme-catalyzed reactions (called the Krebs cycle) in the mitochondrial matrix to generate NADH and FADH_2_. The NADH and FADH_2_ molecules are transferred to the inner mitochondrial membrane and re-oxidized to NAD+ and FAD+ in the electron transport chain (ETC). They donate the carried electrons to the energy receptor (oxygen), formatting ATP through a process called oxidative phosphorylations (OXPHOS).

The ETC consists of five protein complexes forming a redox chain (also known as the respiratory chain). A consequence of electron transport is the production of reactive oxygen species (ROS). Mitochondria-derived ROS include the superoxide anion (O_2_−), hydrogen peroxide (H_2_O_2_), and hydroxyl radicals (OH•) are tightly regulated *via* mitochondrial and cytosolic antioxidant defenses. Superoxide dismutase (SOD), catalase (CAT), glutathione peroxidase (GPX), quinone oxidoreductase 1 (NQO1), and heme oxygenase 1 (HO-1) are enzymes that constitute the key to the cellular antioxidant defense system. Low levels of ROS act as signaling molecules to regulate various intracellular processes (41). However, mitochondrial dysfunction is strongly linked to the excessive release of ROS, which results in oxidative damage to lipids, proteins, and DNA, leading to the development of degeneration and biological aging (42).

Another essential function of mitochondria is to regulate intracellular calcium (Ca^2+^) homeostasis. Mitochondrial Ca^2+^ uptake plays a vital role in regulating cellular functions, involving stimulation of ATP production, inhibition of autophagy, correction of intracytoplasmic Ca^2+^ signaling, and regulation of cell death. Mitochondrial Ca^2+^ uptake is regulated by a multi-protein complex centered around mitochondrial Ca^2+^ uniporter (MCU) and MCU regulatory unit b (MICUb), and in close interactions with several regulatory subunits, such as mitochondrial Ca^2+^ uptake proteins (MICU1 and MICU2) and essential MCU regulator (EMRE). Mitochondrial Ca^2+^ extrusion is mainly dependent on the mediation of Na^+^/Ca^2+^/Li^+^ exchanger (NCLX). Mitochondrial Ca^2+^ overload induces the collapse of mitochondrial membrane potential and the mitochondrial opening of permeability transition pore (mPTP), which triggers the release of pro-apoptotic factors and leads to cell death.

It has been known that mitochondria are closely linked to the inflammatory response. The accumulation of damaged mitochondria leads to the release of several components, such as cell-free mtDNA, N-formyl peptides, and cardiolipin, which can be recognized by pattern recognition receptors (PRRs) as a damage-associated molecular pattern (DAMP) to stimulate inflammation (43). Interestingly, circulating levels of mtDNA rise gradually with age and connect with those of systemic pro-inflammatory cytokines, such as interleukin 6 (IL6) and tumor necrosis factor-alpha (TNF-α) (44).

In addition, mitochondria are involved in a variety of other essential cellular functions, such as the maintenance of ion homeostasis, pH regulation, steroid hormone synthesis, and thermogenesis.

## 3 Mitochondria and sarcopenia

### 3.1 Mitochondrial regulation of sarcopenia

**Figure 1:**
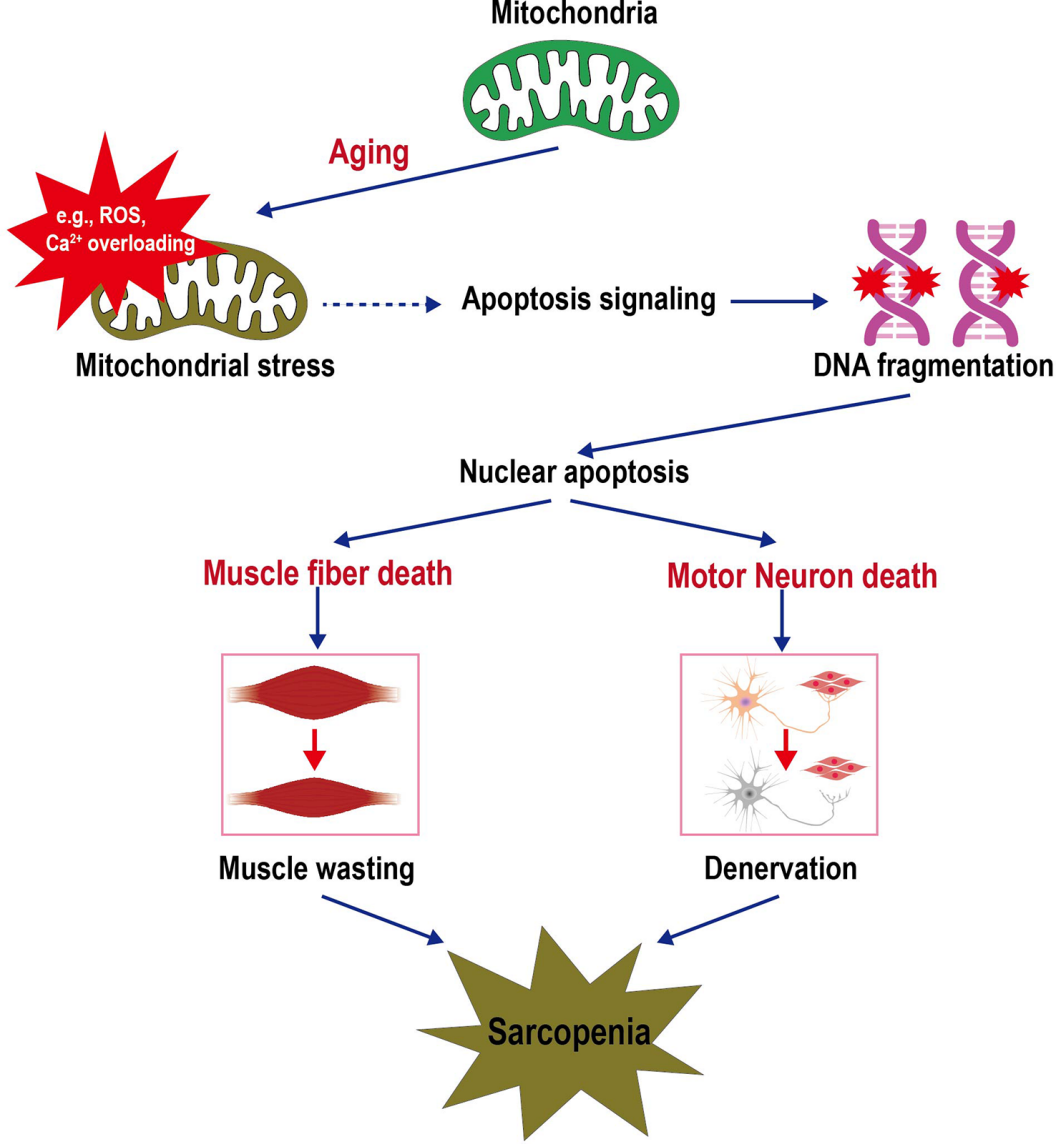
Schematic depicting the sarcopenia triggered by mitochondrial stress in aging. Increased mitochondrial stresses in the aging lead to mPTP opening. The release of mitochondrial contents (e.g., ROS, Ca^2+^, and cytochrome c) into the cell cytosol initiates an apoptotic signaling cascade that culminates in DNA fragmentation and loss of the nucleus (apoptosis). Sufficient nuclear death in a muscle cell will result in the death and removal of the entire muscle cell. Furthermore, motor neuron death coincides with skeletal muscle cell death, i.e., the apoptotic removal of alpha motor neuron nuclei. The death of these cells contributes to the loss of muscle mass and function during aging. Thus, dysfunctional mitochondria trigger a cascade of signals that initiate the signaling pathways that lead to sarcopenia.

Mitochondria play a central role in regulating multiple vital cellular processes involved in energy supply, cellular proteostasis, ROS production, calcium homeostasis, and cell apoptosis (45). Aging increases the levels of mitochondrial stress leading to the increased sensitivity of the mPTP opening. The mPTP is a weakly-selective, large-conductance channel that is closed under non-stressed conditions, which can be triggered to open by mitochondrial ROS (mROS) and Ca^2+^ overloading. Excessive opening of mPTP opening causes the loss of mitochondrial membrane potential and subsequent release of mitochondrial contents (e.g., mROS and cytochrome c) to the cytosol, thereby initiating an apoptotic signaling cascade in muscle fiber and motor neurons. The activation of apoptotic signals is accompanied by DNA fragmentation and subsequent nuclear apoptosis, which eventually leads to muscle atrophy and denervation. **Figure 1** shows the general pathways initiated by mitochondrial alteration resulting in motor neuron and muscle cell death and culminating in sarcopenia (14).

As mentioned above, mitochondria were thought to the main source of ROS production. Conversely, the accumulation of ROS in the muscle and neuron cells has the potential to damage cellular mitochondria (46). This is mainly attributed to the decrease of antioxidant enzymes level in cells caused by aging. For instance, the reduction of the cytosolic antioxidant CuZn-superoxide dismutase (CuZnSOD) was observed in mouse nerves and muscle, consistent with sarcopenic muscle loss (47). In deed, the production of mild oxidative stress acts as a cellular signal that increases skeletal muscle strength, while further increases reduce strength and promote muscle fatigue (48). Considering the age-related decline in cellular antioxidant activity, the accumulation of cellular macromolecular damage induced by free radicals may be a critical driving force for muscle degeneration.

Mitochondrial Ca^2+^ is a key regulator of mitochondrial function and cell death. An increase in Ca^2+^ concentration induces mPTP opening, which further exacerbates the imbalance of intracellular Ca^2+^ homeostasis, possibly caused by leaky ryanodine receptors in aged skeletal muscle (49). Mitochondrial Ca^2+^ overload results in mitochondrial swelling, with perturbation or rupture of the outer membrane, and in turn the release of mitochondrial apoptotic factors into the cytosol. This is partly responsible for the increased incidence of apoptosis in aged skeletal muscle cells (50).

Aging decreases the mitochondrial function and mitochondrial content in the skeletal muscle. As mentioned above, muscle contraction begins with a signal, a neuronal action potential, travels through motor neuron. Neural conduction and the reset of membrane potential after each action potential is a high energy-consuming process. Therefore, decline in mitochondrial function and loss of mitochondrial content in motor neurons may contribute to a decrease in muscle strength (51, 52). Nevertheless, the role of mitochondria in regulating motor neuron aging deficits or motor neuron death is not well understood.

The primary causes of the decline in mitochondrial function and mitochondrial content in aging muscle are the result of the failing mitochondrial quality control (MQC) processes (a series of processes including proteostasis, biogenesis, dynamics, and mitophagy). The following subsections summarize the current evidence supporting MQC derangements as a factor in sarcopenia.

### 3.2 Mitochondrial proteostasis system in sarcopenia

Loss of proteostasis leads to proteome mislocalisation and aggregation, triggers proteotoxic insults and cell death, which further adversely impacts the physiological function of skeletal muscle (53). To maintain the homeostasis of the mitochondrial proteome, mitochondria are equipped with a proteostasis system that consist mainly of a cooperative network of organelle-specific proteases (mitoproteases) and the ubiquitin proteasome system (UPS). A recent proteomics-based study of Drosophila fibroblasts found that approximately 70% of mitochondrial protein turnover occurs through non-autophagic degradation processes such as mitoproteases and UPS; Autophagy accounts for about 30% of mitochondrial protein turnover (54).

Mitoproteases serve as the first line of defense against mild mitochondrial damage and involves the degradation of misfolded or damaged proteins (55). Since about two-thirds of the mitochondrial proteins are located in the mitochondrial membrane and matrix, regulation of the enormously protein-dense matrix environment is particularly important for retaining normal mitochondrial functions. In the mitochondrial matrix, protein turnover is regulated by three core ATP-dependent AAA proteases: Lon protease homolog (LONP1), Clp protease proteolytic subunit (CLPP), and matrix (m)-AAA protease. Several recent studies have suggested a link between AAA proteases-dependent mitochondrial protein quality control and muscle quality/function maintenance. A reduced muscle mass and strength has been reported in mice with deletion of the *Lonp1* gene in response to muscle disuse (56). Down-regulation of ClpP in C_2_C_12_ mouse myoblasts resulted in mitochondrial dysfunction and reduction of cell proliferation (57). Mutation of Drosophila m-AAA mitochondrial protease leaded to impaired mitochondria function, shortened lifespan, and neuronal and muscular degeneration (58). Additionally, protein degradation in the inter-membrane space is mainly achieved by intermembrane (i)-AAA protease YME1L1, High-temperature requirement protein A2 (HTRA2/OMI), the metallopeptidases OMA1 and the presenilins- associated rhomboid-like protein (PARL). Protein quality control in the mitochondrial intermembrane space is also critical to safeguard a proper mitochondrial function and skeletal muscle funciton. Absence of HTRA2/OMI protease activity induced denervation-independent skeletal muscle degeneration with sarcopenia phenotypes in mice (59). Suppression of PARL protein in cultured healthy human myotubes lowered mitochondrial mass and insulin-stimulated glycogen synthesis and increased reactive oxygen species production (60).

The UPS is a primary cytosolic protein degradation system that removes misfolded, damaged, or aging proteins from multiple cellular compartments (61). Components of the ubiquitin system are present in mitochondria, confirming the link between the UPS and mitochondria. The deubiquitinating enzymes UBP16/USP30, E3 ubiquitin ligases Parkin, MITOL/MARCH5, and RNF185 were found in the outer mitochondrial membrane and participate in the regulation of mitochondrial morphology and protein turnover (62–65). The mitochondria-associated degradation (MAD) is similar to the process of endoplasmic reticulum-associated protein degradation (ERAD). In both cases, ubiquitinated proteins are pulled out from organelles by the chaperone AAA-ATPase p97/Cdc48p, subsequently deubiquitinated and degraded by the proteasome in the cytosol (66). p97, also referred to as valosin-containing protein (VCP) or the cofactor Npl4, is a pivotal regulator of protein homeostasis pathways (67). However, additional studies are warranted to understand the role of UPS in degrading mitochondrial membrane proteins and the importance of UPS in sarcopenia.

On the other hand, multiple cellular stresses associated with the aging process trigger the mitochondrial unfolded protein response (UPR^mt^) that serves as a critical mechanism for promoting cellular recovery and mitochondrial network survival (68). Under stress conditions, UPR^mt^ occurs in an attempt to maintain mitochondrial proteostasis by upregulating the expression of mitochondrial chaperones and m-AAA proteases (e.g., chaperonin 10, chaperonin 60, and CLPP) (69). The activation of these signaling pathways improves protein folding, suppresses ER stress, and removes damaged proteins.

### 3.3 Mitochondrial biogenesis in sarcopenia

Fewer mitochondria mass has been observed in skeletal muscle of the elderly compared to young adults (70). This is believed to primarily account for inadequate mitochondrial biogenesis, i.e., a decrease in the production of new mitochondria. PGC-1α, a master transcriptional regulator of mitochondrial biogenesis, has been shown to be reduced at both mRNA and protein levels in aged skeletal muscle (71, 72). PGC-1α activity is co-regulated by phosphorylation and NAD(+)-dependent deacetylation *via* metabolic biosensors AMP-activated protein kinase (AMPK), mitogen-associated protein kinase (p38MAPK), and sirtuin 1 (SIRT1) (73, 74). Once PGC-1α is activated, it powerfully upregulates the expression of several proteins (e.g., NRF1, NRF2, ERRα, ERRβ, ERRγ, TFAM) encoded by both nuclear and mitochondrial genomes, leading to an increase in mitochondrial mass (75).

Recent studies based on the rodent model have revealed a link between biogenesis-related gene expression centered on PGC-1α and skeletal muscle aging. The expression levels of PGC-1α, NRF1, and TFAM are decreased in the skeletal muscle of senescence-accelerated mouse (SAM) prone 8 (SAMP8) during the onset and development of sarcopenia (76). NRF2 knockout exacerbated frailty and sarcopenia of mice during aging, accompanied by the reduced expression levels of PGC-1α, NRF1, and TFAM, as well as a reduction of mitochondrial content in the skeletal muscle (19). Additionally, it has been found that SIRT1 serves as a potential target that is activated by myricanol to increase PGC-1α activity to ameliorate dexamethasone-induced skeletal muscle wasting (77). PGC-1α has also been reported to mediate the beneficial effects of exercise training on aging-related mitochondrial remodeling and muscle functional deterioration in old mice (78). In contrast, overexpression of PGC-1α can mitigate the effects of aging on muscle by increasing mitochondrial protein content and antioxidant enzyme activity and altering gene expression to resemble a youthful transcriptome profile (79, 80). Based on these findings, PGC-1α-mediated mitochondrial biogenesis may be a promising target for sarcopenia therapy.

### 3.4 Mitochondrial dynamics in sarcopenia

As highlighted above, mitochondria are highly dynamic organelles that continually undergo fusion and fission to maintain their morphology, distribution, and function. Fragmented and atypically enlarged mitochondria were often observed in aged skeletal muscle (81, 82), indicating that mitochondrial dynamics are compromised in advanced age. Abnormal mitochondrial morphology and function in aged muscles are accompanied by changes in the expression of fusion and fission proteins, including the fusion factors, MFN1, MFN2, and OPA1; fission factors, DPR1 and FIS1. For instance, reduced expression levels of OPA1 (71) and MFN2 (83) have been described in the skeletal muscle of elderly individuals.

Several recent studies have highlighted the physiological importance of genes encoding fission and fusion machinery components in maintaining skeletal muscle health. The absence of MFN2 in young muscle causes mitochondrial fragmentation, impairs mitochondrial function, enhances ROS production, and promotes the onset of sarcopenia (84). The deletion of OPA1 in the skeletal muscle of young mice also alters mitochondrial morphology and function, leading to muscle loss and weakness (85, 86). Conversely, OPA1 overexpression protects mice from acute and chronic muscle atrophy by ameliorating mitochondrial cristae remodeling and mitochondrial dysfunction (87, 88). Additionally, muscle-specific ablation of DRP1 induces severe muscle wasting and weakness, as well as abnormal morphology, function, and Ca^2+^ homeostasis in mitochondria (18). Another recent study revealed that loss of FIS1 results in impaired mitochondrial function and proteostasis in muscle, decreasing both flight capabilities and lifespan in FIS1 mutant flies (89).

These studies mentioned above indicated the essential role of mitochondrial dynamics in muscle aging and sarcopenia. However, the mechanisms under these processes are still poorly understood. Furthermore, mitochondrial dynamics machinery is closely associated with mitophagy. Mitochondrial fission is necessary for subsequent mitophagy to dissipate dysfunctional parts from the mitochondrial network (90). For example, MFN2 deficiency promotes muscle aging, also reduces autophagy, and leads to the accumulation of defective mitochondria (84). Therefore, further investigation will be needed to clarify the precise molecular mechanisms of mitochondrial dynamics alteration in aging muscle and develop an efficient strategy to prevent or delay the onset of sarcopenia.

### 3.5 Mitochondrial autophagy in sarcopenia

Another primary cause of mitochondrial dysfunction in aging muscle is the impaired mitochondrial autophagy (mitophagy) machinery. Mitophagy is a particular type of macroautophagy (autophagy) and, as mentioned above, can selectively remove dysfunctional and damaged mitochondria to maintain mitochondrial homeostasis. Studies indicate that aging provokes certain alternations of several key mitophagy regulators, leading to mitophagy deficiency and subsequent accumulation of dysfunctional mitochondria in skeletal muscle (91, 92).

Among mitophagy proteins, PINK1 and Parkin (PARK2) have been identified as crucial components in response to mitochondrial damage (93). loss of Parkin causes a decline in muscle force in mice, as well as impaired mitochondrial respiratory function and increased sensitivity to mPTP in skeletal muscle (94). In line, overexpression of Parkin attenuates aging-related loss of muscle mass and strength, along with improved mitochondrial biogenesis and enzymatic activities (95). In addition, several studies have revealed the complexities and interrelationships between the different pathways for maintaining mitochondria fitness. PINK1 can serve as a pro-fission signal by activating DRP1 in response to mitochondria damage (96). Parkin inhibits refusion after mitochondrial fission upon depolarization by inducing the proteasomal degradation of mitofusins (97). Besides, PGC-1α overexpression reduced the expression of PINK1 and Parkin in muscle disuse atrophy (98). Alternatively, MFN2 deficiency in muscle during aging reduces autophagy, which contributes to the accumulation of damaged mitochondria and triggers age-related mitochondrial dysfunction (84).

These findings indicate that mitophagy may contribute to sarcopenia through a complex MQC network. Notably, mitochondrial-derived vesicles (MDVs) have also been identified as a novel way that eliminates damaged mitochondria alternatively of mitophagy (99). Circulating MDV-derived ubiquinone oxidoreductase subunit S3 (NDUFS3), the reduced form of nicotinamide adenine dinucleotide, may be a novel predictor of sarcopenia (100). Thus, the maintenance of mitochondrial homeostasis in aging muscles is coordinately regulated by multiple pathways.

## 4 Role of 17β-estradiol and testosterone on mitochondria

### 4.1 Sexual dimorphism of mitochondria

Although mitochondria are inherited maternally, as mentioned above, almost all mitochondrial proteins are encoded in the nucleus and are therefore influenced by sex chromosomes and circulating sex hormones. Increasing evidence suggests mitochondria are highly tissue and sex-specific in males and females. For instance, female mitochondria in the heart, liver, and brain have a higher antioxidant capacity and produce less ROS than males (101, 102). Similarly, females have more functional mitochondria content in white and brown adipose tissue than males (103, 104). Conversely, the female brain and heart mitochondria have lower Ca^2+^ uptake than males (105, 106).

Additionally, previous studies have revealed that female rodents show higher endurance capacities and anti-fatigue properties than male rodents (107, 108). This is believed to be associated with mitochondrial content and substrate utilization in skeletal muscle. Compared with males, the gastrocnemius muscle of female rats exhibits higher mitochondrial DNA/nDNA and OXPHOS capacity (109). Moreover, female skeletal muscle has higher intracellular lipid content than males and thus may depend on fat oxidation to supply much more energy during exercise (110).

Other aspects of sex differences in mitochondria in different tissues include mitochondrial biogenesis, autophagy, susceptibility to mPTP opening, and cell apoptosis (111–113). Undoubtedly, more systematic investigation for sex dimorphism would contribute to further understanding the role of mitochondria in the sex specificity of important pathologies, such as sarcopenia.

Although sexual dimorphism in mitochondrial function have been related to the genetic interactions between sex chromosomes and autosomes (114, 115). The more recent data, however, suggest that sex hormones contribute to this sex specificity on mitochondria. The effects of estrogen and androgens on mitochondria will be discussed below.

### 4.2 Role of 17β-estradiol on mitochondria

17β-estradiol is the most potent and ubiquitous member of the category of sex steroid hormones known as estrogen, primarily synthesized in the ovaries from cholesterol. It can also be produced locally from fat, brain, skeletal muscle, and testes by aromatization, which converts androstenedione and testosterone to 17β-estradiol (116, 117). The biological effects of estrogen are mainly mediated *via* nuclear and membrane estrogen receptors (ERs), including estrogen receptor α (ERα), estrogen receptor β (ERβ), and G-protein-coupled ER (GPR30 or GPER), all three bind 17β-estradiol with high affinity in the low nM range (118, 119). 17β-estradiol binds to ERs to regulate gene expression through genomic and non-genomic mechanisms. In the genomic pathway, cytosol-localized ERα and ERβ act as nuclear transcription factors that typically dimerize and translocate to the nucleus following ligand binding to activate gene transcription and expression. In the non-genomic pathway, membrane-localized ERα and ERβ sub-population, as well as GPER, trigger various protein-kinase (MAPK, PKB, and PKC) cascades. A more detailed description of the genomic and non- effects of Estrogen/ERs can be found in ref (120).

Accumulating research shows estrogen can affect mitochondrial mass and function through both genomic and non-genomic pathways. For instance, estrogens upregulate the expression of PGC-1 and its downstream target genes *via* genomic ERα and ERβ to promote mitochondrial biogenesis and ATP production (121). 17β-estradiol also increases the transcription of mitochondrial nuclear-encoded genes, and mitochondria-encoded genes through the ERα/β mediated activation of NRF1 and TFAM (122, 123). On the other hand, it has been reported that ERα and GPER mediate 17β-estradiol enhancement of mitochondrial respiratory capacity and ATP production *via* a PKA-dependent mechanism (124). Activation of GPER is associated with the inhibition of mPTP opening, an effect mediated by the ERK pathway (125). Moreover, 17β-estradiol also appears to regulate multiple other aspects of mitochondrial function through ERs, including ROS generation, antioxidant defense, and Ca^2+^ handling (126–131). Interestingly, it was found that subpopulations of ERα and ERβ exist in mitochondria, although it is still unclear if they can directly regulate mtDNA transcription (132–134). A recent study also showed that 17β-estradiol could directly reduce the microviscosity of mitochondrial membrane and bioenergetic function in skeletal muscle without dependence on its receptor (135). For a detailed description of the regulation of estrogens on mitochondrial function, see (136, 137).

### 4.3 Role of testosterone on mitochondria

Along with 17β-estradiol, testosterone plays an essential role in skeletal muscle physiology. Testosterone is a representative sex steroid hormone, mainly produced by male Leydig cells, female ovarian thecal cells, and partly by the adrenal gland (138). In the cell cytoplasm, testosterone is converted into its active form, dihydrotestosterone (DHT), by 5α-reductase. The actions of testosterone are mainly mediated by androgen receptor (AR), which binds to specific androgen response elements (AREs) in the promoter regions of target genes (139). As a principal anabolic hormone, testosterone plays a crucial role in increasing protein synthesis and inhibiting muscle proteolysis in skeletal muscle (140, 141). Additionally, testosterone can promote muscle fiber regeneration and repair by activating muscle satellites and increasing cellular insulin-like growth factor-1 (IGF-1) levels (142, 143).

Similar to 17β-estradiol, testosterone can also influence mitochondrial function in several ways, including mitochondrial biogenesis, mitophagy, and mitochondrial ATP production. It has been reported that knockout of AR results in the down-regulation of PGC-1α and TFAM in the muscle of castrated rats and mice, while the administration of exogenous androgen reversed these effects (144, 145). Castration leads to a decrease in mtDNA copy number in the skeletal muscle of male pigs, suggesting that testosterone is required to maintain mitochondrial copy number (146). In addition, Castration increases LC3 II/I ratio in the skeletal muscle of male mice, indicating that androgen deficiency increases mitophagy (147, 148). These studies suggest that androgens may maintain mitochondrial mass by inducing mitochondrial biogenesis and inhibiting autophagy. Moreover, testosterone may protect the respiratory chain of mitochondria from oxidative damage and maintain a normal OXPHOS function (149). Notably, Similar to the Localization of estrogen receptors (ERα and ERβ), a recent study has shown that besides being nuclear, AR also localizes into mitochondria (150). However, little is known about the role of AR in mitochondrial Localization, and further studies are needed to elucidate the underlying mechanisms.

## 5 Mitochondria: a central target for 17β-estradiol and testosterone in age-related muscle degeneration

Based on the information discussed, mitochondria are crucial for skeletal muscle to maintain normal metabolic function and respond to physiological or pathological stimuli. Age-related muscle degeneration is caused when mitochondria are defective or abnormal and cannot be cleared or degraded effectively. Multiple factors, including dysregulation of mitochondrial OXPHOS, mutation or deletion of mtDNA, altered expression or function of mitochondria-associated proteins, imbalanced Ca^2+^ homeostasis, and ultrastructural defects, may cause mitochondrial dysfunction.

**Figure 2:**
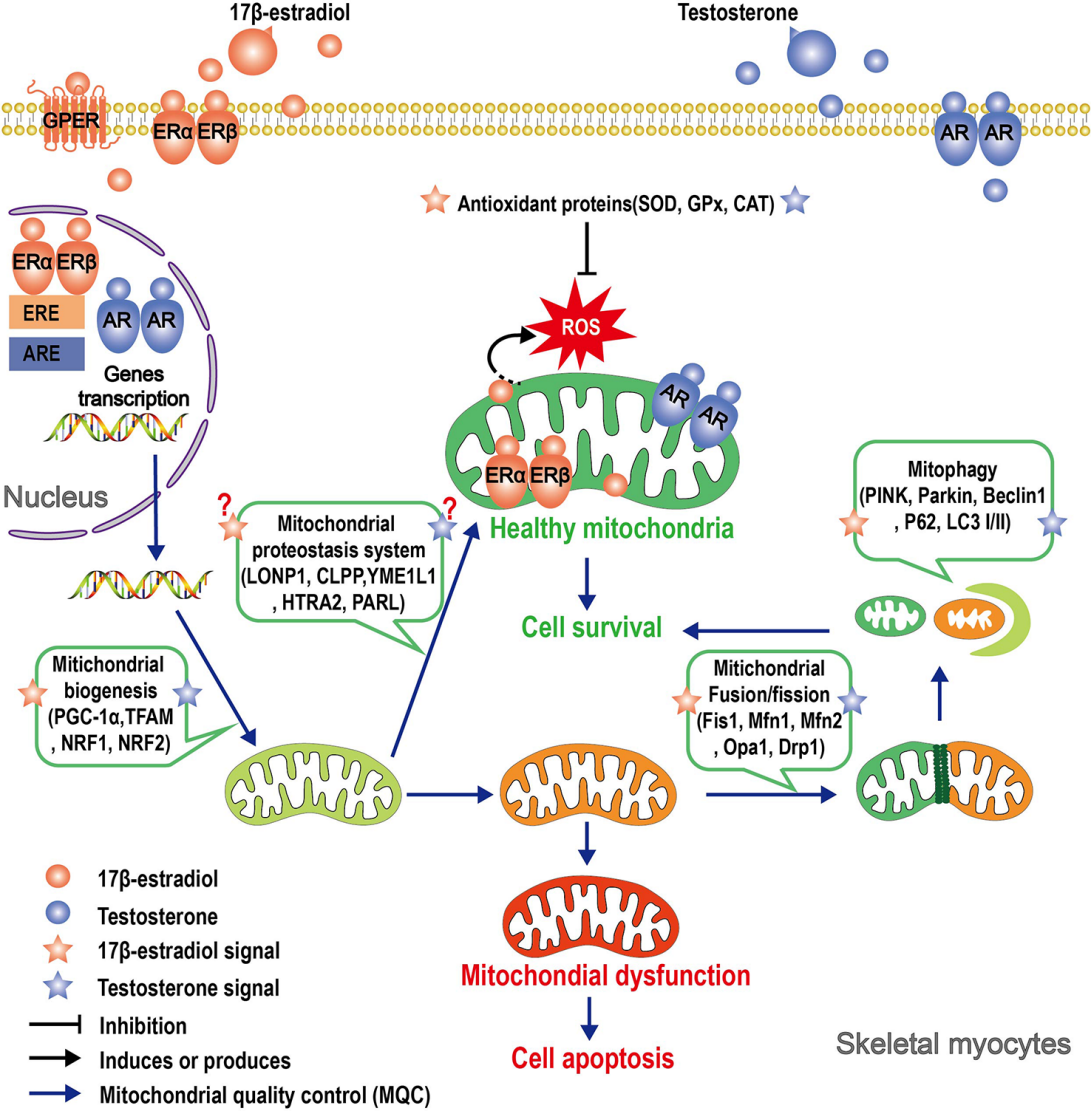
Schematic illustrating the roles that 17β-estradiol and testosterone play in mitochondrial protection of skeletal myocytes. Signals of 17β-estradiol and testosterone affect mitochondria function through multiple pathways. In the genomic pathway, 17β-estradiol or testosterone binds to its receptor, thereby inducing receptor dimerization and translocation of the entire complex to the nucleus. In the nucleus, the dimer of the receptors binds to estrogen response elements (ERE) or androgen response elements (ARE) and affects the transcription of nuclear-encoded mitochondrial genes. ERs and AR have been shown to localize in mitochondria, but it is yet unclear if the complex can directly regulate the transcription of mtDNA-encoded genes. The “nongenomic pathway” involves rapid activation of various kinases by membrane-associated ERs or AR, which in turn can affect mitochondrial function. Both 17β-estradiol or testosterone regulate different parameters of MQC, such as proteostasis, biogenesis, dynamics, and mitophagy. A consequence of the action of these sex hormones is mitochondrial protection, although the specific mechanism of action has not yet been elucidated.

The decline in mitochondrial function and the reduction in circulating 17β-estradiol and testosterone that accompany aging may be two closely related processes. Obviously, both 17β-estradiol and testosterone exert actions on mitochondria: 17β-estradiol and testosterone act directly on mitochondria, also through ERs and AR located in the organelle, and indirectly regulate nDNA-encoded mitochondrial proteins and nuclear transcription factors that affect mtDNA-encoded proteins. Similarly, these sex hormones indirectly control various mitochondrial functions, such as ROS production and apoptosis, through modulation of plasma membrane receptor-induced kinase signaling pathways or through cytosolic signal peptides. Both steroids trigger complex molecular mechanisms involving crosstalk between mitochondria, the nucleus, and the plasma membrane, and the result of this action is mitochondrial protection (**Figure 2**). Therefore, the molecular components of the pathways activated by the sexual steroids be putative targets for anti-muscle decay strategies.

Accumulating evidence reveals that 17β-estradiol and testosterone play important roles in regulating mitochondrial homeostasis in skeletal muscle. However, controversy remains regarding indications for exogenous 17β-estradiol or testosterone supplementation in aging people due to a shortage of clinical trials demonstrating these steroids’ benefits and adverse effects. Data from the Women’s Health Initiative (WHI) shows several risks of menopausal hormone therapy among healthy postmenopausal women, including an excess risk of coronary heart disease, stroke, and breast cancer (151). In this context, oral intake of natural food sources containing phytoestrogens may be a good alternative. For example, intakes of soymilk containing isoflavones, an active estrogen-like substance, improve muscle weakness in OVX mice (152). Similar to 17β-estradiol replacement treatment, several investigators found the potential risks associated with androgen therapy (153, 154). However, these researchers reported that low-intensity physical exercise combined with exogenous testosterone supplementation improves grip strength, spontaneous movement, and breathing activity (155). Interestingly, our previous studies have shown that exercise training can upregulate the expression of aromatase in rat skeletal muscle and increase the level of 17β-estradiol in skeletal muscle, which may be related to exercise improving skeletal muscle mass in OVX rat (117). Therefore, a better understanding of the role of endogenous hormones in skeletal muscle may be a direction worthy of attention in future research.

## 6 Conclusions

Overall, sarcopenia is a complex geriatric condition associated with multiple negative health-related outcomes, such as frailty, hospitalization, and mortality. However, despite the efforts made towards development, to date, there are still no authorized drugs for managing sarcopenia (156). Evidence suggests that sarcopenia can be prevented and possibly reversed through lifestyle interventions such as resistance exercise and proper nutritional support (157, 158). While neither exercise nor nutrition can entirely treat the age-associated decline in muscular mass and strength, it can certainly slow down the development and reduce the rate of sarcopenia (158). Nonetheless, there is a rising interest in clinical trials for drug treatments aimed at reducing sarcopenia. These treatments focus on enhancing muscle mass by administering testosterone injections, selective androgen receptor modulators (SARMS), and growth hormones, while efficacy and safety are still being investigated (159). Obviously, additional work is needed before we completely understand the etiology of sarcopenia.

We contend that mitochondrial-regulated apoptosis is central to initiating the signal for age-related skeletal muscle deterioration. The impaired mitochondrial function and its associated mitochondrial signaling events activate apoptotic signaling in muscle cells and motor neurons. Therefore, it is crucial to maintain the health and functionality of mitochondria during aging. This largely depends on effective MQC machineries such as proteostasis, biogenesis, dynamics, and mitophagy, which work together to ensure that damaged or dysfunctional mitochondria are eliminated and replaced with healthy ones. We outlined that errors in any MQC process steps could ultimately contribute to the age-associated decline of muscle mass and function.

It is becoming increasingly clear that estradiol and testosterone co-regulate mitochondrial biogenesis, dynamics, and autophagy to maintain mitochondrial function in skeletal muscle. We propose that age-related decline in both sex hormones may trigger sarcopenia by initially impairing mitochondrial function rather than being an independent factor. A comprehensive understanding of the molecular mechanisms elicited by both steroids at the mitochondrial level and their effects on skeletal muscle mass and function will lead to a better understanding of sarcopenia and facilitate more appropriate therapeutic interventions.

## Author contributions

RS and SL conceived the idea. XT wrote the original manuscript. RS and SL revised the manuscript. All authors contributed to the article and approved the submitted version.
